# Frequency of null allele of Human Leukocyte Antigen-G (HLA-G) locus in subjects to recurrent miscarriage

**Published:** 2016-07

**Authors:** Nazila Alizadeh, Elnaz Mosaferi, Laya Farzadi, Jafar Majidi, Amir Monfaredan, Bahman Yousefi, Behzad Baradaran

**Affiliations:** 1 *Immunology Research Center, Tabriz University of Medical Sciences, Tabriz, Iran.*; 2 *Women Reproductive Health Research Center, Tabriz University of Medical Sciences, Tabriz, Iran.*; 3 *Department of Hematology, Faculty of Medicine, Tabriz Branch, Islamic Azad University, Tabriz, Iran.*; 4 *Students Research Committee, Tabriz University of Medical Sciences, Tabriz, Iran.*; 5 *Nazila Alizadeh and Elnaz Mosaferi contributed equally to this work and both are first author.*

**Keywords:** *Allele*, HLA-G*0105N, *Recurrent miscarriage*, *RFLPs*

## Abstract

**Background:**

Human leukocyte antigen-G (HLA-G) is a non-classical class I molecule highly expressed by extravillous cytotrophoblast cells. Due to a single base pair deletion, its function can be compensated by other isoforms. Investigating the frequency of null allele in Recurrent Miscarriage (RM) subjects could be useful in understanding the relationship between frequency of this allele and RM in a given population.

**Objective:**

This study aimed to determine the frequency of HLA-G*0105N null allele and its potential association with down-regulation of HLA-G in subjects with RM.

**Materials and Methods:**

Western blotting was used to assess the level of HLA-G protein expression. For investigating the frequency of HLA-G*0105N null allele in RM subjects, PCR-RFLP method was used. Exon 3 of HLA-G gene was amplified by polymerase chain reaction (PCR). Subsequently, PpuM-1 enzyme was employed to digest the PCR products and fragments were analyzed using gel electrophoresis.

**Results:**

Digestion using restriction enzyme showed the presence of heterozygous HLA-G*0105N null allele in 10% of the test population. Western blotting results confirmed the decrease in expression of HLA-G in the placental tissue of subjects with RM compared to subjects who could give normal birth.

**Conclusion:**

The frequency of heterozygous HLA-G*0105N null allele was high to some extent in subjects with RM. The mutation rate in subjects suggested that there is a significant association between RM and frequency of mutations in this allele.

## Introduction

Human leukocyte antigen-G (HLA-G) is known to have an essential role in protecting semi-allogeneic fetus against maternal immune responses and is a non-classical tissue specific class I molecule which is highly expressed on extra villous cytotrophoblast cells (1, 2). HLA-G is located in the major histocompatibility complex (MHC) at 6p21.3, which is one of the most polymorphic regions within human genome (3). Although classical HLA-G class I molecules polymorphism is centralized around the peptide binding groove, the HLA-G polymorphism is usually located between the α1, α2 and α3 domains (4). 

The HLA-G gene undergoes alternative splicing, resulting in production of four membrane-bound (HLA-G1_G4) and three soluble (HLA-G5_G7) proteins. Both membrane-bound and soluble isoforms of HLA-G have the capability to inhibit NK cell and antigen-specific T cell cytotoxicity and cells also to block the proliferation of allogeneic T cells, as shown by functional assays. Furthermore, soluble HLA-G isoforms can promote apoptosis of NK and activated CD8^+^ T cells (5-11). 

Being mainly expressed on invasive trophoblastic cells, HLA-G is the most important factor in maternal-fetal immune tolerance. The HLA-G gene contains 15 alleles, including the HLA-G*0105N null allele which presents a single base pair deletion in exon 3 (12). The deletion of cytosine at codon 130 disrupts the open reading frame, and eventually inhibits the translation of HLA-G1 and G5. However, this null allele retains its ability to translate both the membrane bound HLA-G2 and HLA-G3 as well as the soluble HLA-G6 and HLA-G7 isoforms. The discovery of normal healthy individuals homozygous for HLA-G*0105N allele refers to the fact that the other HLA-G isoforms encoded by this allele have the ability to compensate for the lack of both HLA-G1 and HLA-G5 proteins and retaining the fetal-maternal immune tolerance (13, 14). Considering the results from studies on the frequency of HLA-G*0105N null allele in Iranian population and other populations by Rahimi *et al*, showed that the frequency of this allele is higher in Iranian healthy subjects than other ethnic groups (15).

This study was designed to investigate the frequency of HLA-G*0105N null allele in subjects with recurrent miscarriage (RM) in East Azerbaijan, Iran.

## Materials and methods

**Population**

In this case-control study, the first step to find the relation of HLA-G*0105N null allele and RM was to determine the frequency of this allele with PCR-RFLP method. For this reason recurrent pregnancy loss (RPL) samples were collected. Sample collection was performed during February to March of 2014 in Tabriz which is a major city in East Azerbaijan province. This study was approved by the ethical committee of Tabriz University of Medical Sciences and the written consent form was obtained from all participants.

For this purpose, 60 whole blood samples of subjects with RM were collected. Also following previous studies to confirm down regulation of HLA-G protein with immunoblotting method, 20 placental tissue samples of RM patients and 20 placental tissue samples of normal pregnancies (as control) were randomly selected from maternity hospitals in Tabriz. 

Inclusion criteria for this study included women with history of RM (two or three times or more, continuously and under twenty weeks) and exclusion criteria included trauma, chromosomal abnormalities, induced abortion and infections. 

**DNA extraction**

Genomic DNA was extracted from the whole blood samples collected in EDTA-containing vacationer tubes using salting-out method (16). DNA concentrations and purity was determined by biophotometer and Gel electrophoresis (1.5% agarose).

**PCR-RFLP analysis**

HLA-G genotyping was performed using the polymerase chain reaction- restriction fragment length polymorphism (PCR-RFLP) analysis. PCR reaction was performed for HLA-G polymorphisms. Cycles were consisted of: initial denaturation at 95^o^C for 5 min, 35 cycles 94^o^C for 30 sec, 63^o^C for 45 sec, and 72^o^C for 30 sec. The final cycle was followed by an extension step of 300 sec at 72^o^C. For the detection of the polymorphic HLA-G restriction enzyme site, 2 primers were used: F5´-CAC ACC CTC CAG TGG ATG AT-3´ and R5´-GGT ACC CGC GCG CTG CAG CA-3´. PCR products were checked by electrophoresis on 2% agarose gel. 

PCR products were digested with PpuM-1 restriction enzymes at 37^o^C for overnight according to manufacturer’s instructions (Fermentas M Medical Srl, MD, USA). The RFLP products were analyzed using electrophoresis on 1.5% agarose gel, then were stained with ethidium bromide and visualized under shortwave ultraviolet light, then genotype was determined. Lack of the restriction site for the PpuM-1 enzyme in the exon 3 of HLA-G was illustrated for the presence of HLA-G*0105N allele (17).

**Western blot analysis**

Expression of HLA-G protein was investigated by Western blotting. Equal amount (25 μg) of total protein from each tissue samples was heated for 5 min at 95^o^C before loading and separated on 12.5% SDS polyacrylamide gels using a mingle apparatus (Bio-Rad Laboratories). Proteins were then transferred to polyvinyl denied fluoride (PVDF) membranes (Millipore; Billerica, MA). 

Following on, membranes were blocked with 5% nonfat dry milk in TBS/Tween-20 (0.05%, v/v) for 1 hr at room temperature and then were incubated overnight at 4^o^C with the appropriate primary antibodies [anti-HLA-G and β-Actin (Abcam, Cambridge, MA, UK)] in TBS/T buffer (in a 5:1,000 dilution) followed by incubation with the appropriate HRP-conjugated secondary antibody (1:1,000 dilution; Abcam) for 2 hr at room temperature. After washing, protein bands visualized using enhanced chemiluminescence detection Kit (GE Healthcare) and autoradiography films (Fuji Photo Film Co., Ltd., Tokyo, Japan) according to the manufacturer’s instruction. 

**Statistical analysis**

Statistical analysis was conducted using SPSS 15.0 software (Statistical Package for the Social Sciences, version 15.0, SPSS Inc, Chicago, Illinois, USA) and allele frequencies were determined. The differences in allelic and genotypic frequencies between case and control groups were evaluated by ^2^ test with odds ratio (OR). P<0.05 was considered significant.

## Results

**Figure 1:**
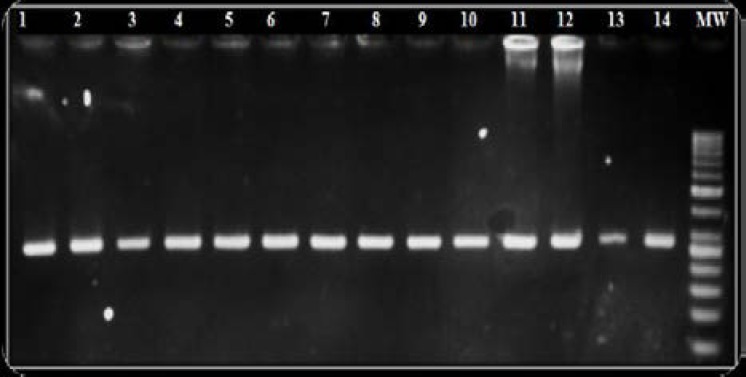
276 bp HLA-G exon 3 PCR products: lanes 1 to 14 are PCR products and lane 15 shows the 50 bp DNA ladder.

**Figure 2:**
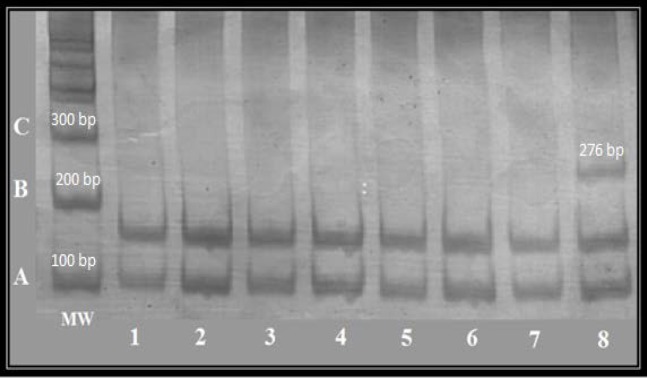
PCR-RFLP analysis of HLA-G* 0105N null allele in subjects with RM. The PCR products were digested overnight with PpuM-1 restriction enzyme. The lane MW represents the 100 bp DNA ladder. Lanes 1 – 7 show products after restriction digestion with normal pattern (108 and 168 bp). Lane 8 shows the product after digestion with heterozygous pattern (108, 168 and 276 bp).

In this study, investigating the frequency of this allele in RM patients, first the 276 bp fragment containing the desired mutation (HLA-G*0105N), was amplified. Then PCR reaction was optimized (Figure 1). PCR-RFLP results are shown in Figure 2. The HLA-G*0105N null allele was distinguished according to the absence of the restriction enzyme product. 

**Figure 3:**
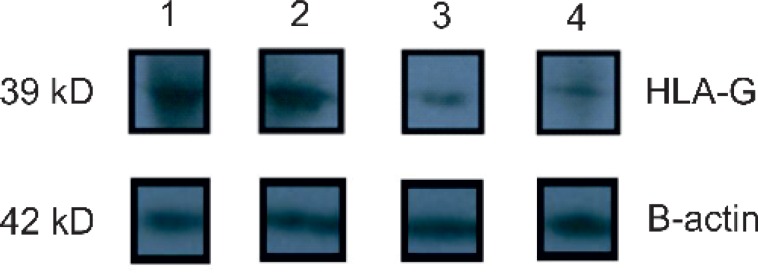
HLA-G protein western blotting shows a specific reduction of the protein levels in the RM group compared with the control group. HLA-G protein is 39 kDa and β actin is 42 kDa. (Number 1 and 2 show control group, while number 3 and 4 show the group with RM).

The mutation of HLA-G*0105N null allele was investigated in 60 individuals with RM. This mutation was recognized on several chromosomes and the frequencies have been presented in Table I. The difference in expression levels between subjects having HLA-G with null mutation and those without null mutation is presented in Table II. The results of western blotting confirmed a decrease in expression of HLA-G in the placental tissue of subjects with RM compared to subjects who could give normal birth (Figure 3). Overall, PCR method combined with RFLP analysis for a specific restriction site of HLA-G gene in exon 3. Six subjects out of sixty were found to be heterozygous for this gene.

**Table I:** The frequency of mutations in the two groups with and without null allele

**Haplotype**	**Frequency**	**SE**	**Frequency**		^2^** value**	**p-value**
			**Controls**	**RPL** *		
CC	0.891	0.026	0.531	0.32	21.452	0.831
- / C	0.109	0.019	0.369	0.68	0.001	0.034

SE: standard Error

* RPL= Recurrent pregnancy loss

**Table II:** Comparison of HLA-G gene expression in presence and absence of null allele mutation

**Group**	**Reaction Efficiency**	**Expression**	**Standard Error**	**95% confidence interval**	**Result**
HLA-G with null mutation	0.97	0.099	0.056 - 0.197	0.024 - 0.286	DOWN
HLA-G without null mutation	0.97	1.171	0.845 - 1.921	0.573 - 2.366	UP

## Discussion

HLA-G gene which plays an important role in normal pregnancy is consisted of 15 alleles. This gene includes HLA-G*0105 N null allele. HLA-G gene function has been implicated in a number of diseases such as cancer and in pregnancy outcome (18-20). HLA-G consists of 15 alleles, including the HLA-G*0105N null allele which is characterized by a single nucleotide deletion and subsequent frame shift mutation in exon 3. Since some healthy women with normal pregnancies heterozygous for the null allele have been observed, HLA-G*0105N null allele is believed to be capable of producing the other HLA-G isoforms such as HLA-G2, HLA-G3, HLA-G6 and G7, which can hypothetically compensate for the lack of HLA-G1 and G5 isoforms. Therefore, the immune tolerogenic role of HLA-G could be accepted as true. Although this allele is shortened, it can still perform some functions like providing a leader peptide for expression of HLA-E (21). 

Since HLA-G*0105N null allele could retain the expression of HLA-G functional proteins, the privilege of the selection of this allele must be investigated more in different populations. The frequency of exon 3 deletion has been already studied in different ethnic groups: 11% in the Shona ethnic group of Zimbabwe, 7.4-8% in African-Americans, 4.8% in Ghanaians, 3% in Spaniards, 2.3% in mixed German-Croatians and 0.6% in Danish populations.

Beside, although the existence of HLA-G*0105N allele has been reported in different populations, in Japanese or American Caucasian populations, there has not been found any HLA-G*0105N allele. Furthermore, the HLA-G*0105N allele is relatively frequent allele in African-Americans (7.4%), meanwhile it has lower frequency in Northern (0.6%) and Middle Europeans (2.3%). In Amerindians, from the Brazilian Amazon region there was not any copy of this allele by exon 3 sequencing (22-28). Above all, the frequency of this allele in Iranian populations was higher than those of other ethnic groups (20%) (15). 

Mendes-Junior *et al* have reported that the frequency of HLA-G*0105N was higher in the areas with high pathogenesis. Due to the proposed function of HLA-G in placenta development, there must be an inverse association between HLA-G1 expression and uteri infection, such that, when HLA-G1 is down regulated in a HLA-G*0105N heterozygous placenta, this may enhance the intrauterine defense against infections (28). Statistical researches which are performed in different races with various genotypes, could represent genetic diversity and appearance of different polymorphic features affecting pathogenesis of distinct illnesses. Decrement of mutation of HLA-G gene in perused statistical society, could mean relation of present mutation and down regulation of this gene. 

The current study aimed to investigate the frequency of null allele and possible association of this mutation with RM. Researches presented that abundance of this allele among Iranian populations is more in comparison with other races which has been studied before (15). HLA-G*0105N null allele which exists in East Azerbaijan population like other parts of Iran. Results demonstrated that HLA-G*0105N null allele occurs at a rate of 10% in subjects with RM in East Azerbaijan population. Also the findings of Naghavian and colleagues in Mazandaran confirmed this study’s findings which showed 59% of women with RM in Mazandaran have had HLA-G*0105N heterozygous allele (29). 

The mutation rate in female subjects also suggested that there is a significant association between RM and frequency of this mutation. So far, different studies have revealed that the frequency of HLA-G*0105N null allele differs among different geographic regions.

However, the variety of heterozygous mutations of this allele in RM subjects in different populations is still unknown. As a suggestion, importance of this allele in different populations and its potential link to normal or pathological pregnancies could be studied further. The present research team had futuristic insight goal, which means if the clinical findings performs as expected, it could be an applicable justification for Iranian female society.

## Note

***This article extracted from M.Sc. thesis. (Nazila Alizadeh)***
